# Manuscript: Defining Quality Standards for Intensive Home Based Treatment Programs for Youth with Serious Emotional Disorders

**DOI:** 10.1007/s10488-021-01116-8

**Published:** 2021-07-09

**Authors:** Eric J. Bruns, Philip H. Benjamin, Richard N. Shepler, Marianne Kellogg, Hunter Pluckebaum, Joseph L. Woolston, Kelly English, Michelle D. Zabel

**Affiliations:** 1 Department of Psychiatry & Behavioral Sciences, University of Washington, 6200 NE 74th Street, Building 29, Suite 110, Seattle, WA 98115 USA; 2 Center for Innovative Practices, Case Western Reserve University, 11402 Bellflower Road, Cleveland, OH 44106 USA; 3 Yale University School of Medicine, Child Study Center, 230 South Frontage Road, New Haven, CT 06519-1124 USA; 4 Children’s Behavioral Health Knowledge Center, Massachusetts Department of Mental Health, 25 Staniford Street, Boston, MA 02114 USA; 5 School of Social Work, University of Maryland, 306 W. Redwood Street, 2nd Floor, Baltimore, MD 21202 USA

**Keywords:** Child, Adolescent, Behavioral health, Treatment, Quality, Fidelity

## Abstract

Intensive Home Based Treatment (IHBT) is a critical component of the continuum of community-based behavioral healthcare for youth with serious emotional disorder (SED) and their families. Yet despite being used nationwide at costs of over $100 million annually in some states, a well-vetted, research-based set of quality standards for IHBT has yet to be developed. The current project aimed to define program and practice standards for IHBT, drawing upon literature review, expert interviews, and a systematic *Delphi* process engaging over 80 participants, including IHBT developers, experts in evidence-based youth mental health, youth and family advocates, IHBT providers, and state policymakers. After two rounds of quantitative and qualitative input, adequate consensus was achieved on 32 IHBT Program Standards and 43 IHBT Practice Standards. These standards hold potential for informing efforts such as development of state regulations, provider contracts, memoranda of agreement, and training and workforce development initiatives. Translation of the quality standards into measurement strategies holds potential for providing a method of continuous quality improvement across multiple levels as well as use in research on IBHT.

## Introduction

Ten percent of all U.S. youths experience serious emotional disorder (SED), defined as a psychiatric disorder that causes substantial impairment in one or more functional domains (Friedman et al. 1996; Williams et al. 2018). Since the publication of *Unclaimed Children* (Knitzer and Olson 1982) and *A System of Care for Children and Youth with SED* (Stroul and Friedman 1986), a primary goal of public serving systems has been to provide children and adolescents with SED access to effective community-based services that can prevent unnecessary out-of-home placement.

By some metrics, this movement has achieved modest success. For example, there has been a proliferation of states that organize some amount of their treatment for youth with SED on community-based systems of care and/or “wraparound” frameworks (Bruns et al. 2011; Sather and Bruns 2016). Evaluations of federal demonstration projects (e.g., Urdapilleta et al. 2011), state and county studies (e.g. Kamradt et al. 2008; Rauso et al. 2009; Yoe et al. 2011), and meta-analyses of controlled research (e.g., Suter and Bruns 2009) on these models have found reductions in institutional care and subsequent costs. Meanwhile, at a population level, Case et al. (2007) found a decline in inpatient mental health treatment of children and adolescents in U.S. community hospitals between 1990 and 2000. In the child welfare sector, the number of foster children in group homes and institutions experienced steady decline until 2012, when rates began to increase again (Child Trends Databank 2018).

Despite evidence of gains, however, there remains a persistent “imbalance” in the children’s behavioral health system (Cooper et al. 2008). Ringel and Sturm (2001) found that approximately one-third of the $12 billion spent on child mental health services were for out-of-home care, and there is little evidence that this imbalance has shifted. Soni (2014) found that mental health disorders represented the most costly health condition of childhood, due primarily to the reliance on institutional care for youths with SED. Pires et al. (2013) have documented that 38% of all Medicaid spending on children’s behavioral health is allocated to the 9% of youth with the most serious needs, mostly due to spending on residential and group placements for these children and youth. Meanwhile, child welfare out-of-home care expenses are estimated to be $10 billion annually, and over $5 billion is spent in juvenile justice to provide residential placements for committed youth (Scarcella et al. 2004; Justice Policy Institute 2009). Given the majority of youth in these sectors have mental health diagnoses (dosReis et al. 2005; Vincent et al. 2008), it is reasonable to assume that a large portion of placements for youth in the child welfare and justice sectors are related to presence of behavioral health needs.

### Intensive Home-Based Treatments

To adhere to federal laws (e.g., the Early and Periodic Screening, Diagnostic and Treatment child health component of Medicaid; *Olmstead v. L.C.* 1999; The Americans with Disability Act of 1990), families and communities need to have access to effective community-based treatment alternatives that reduce the need for more restrictive residential treatment options for youth with SED. Typically, IHBT programs are considered the most intensive community-based treatment option in a comprehensive continuum of care for youth at risk of placement or returning home from placement.

Youth and families requiring IHBT services have multiple and complex needs that require access to an array of services and supports. IHBT is a comprehensive service that offers a range of individual treatment elements integrated into a single coordinated service. These elements typically include: (1) crisis response, stabilization, and safety planning; (2) psychoeducational skill building with youth; (3) skill building with caretakers (parenting and behavior management); (4) cognitive and emotional coping with a focus on trauma-informed care; (5) family systems therapy; and (6) resilience and support-building interventions (Kaplan and Girard 1994; Stroul 1988). The ultimate goal for IHBT is to reduce the risk of out-of-home placement for the youth, through (1) reduction in the youth’s emotional and behavioral symptomatology; (2) creation of safe, trauma-free environments; (3) improvement in family communication and relationships; (4) reduction in the magnitude and frequency of family conflicts; (5) an increase in youth and family supports, resources, and resilience; (6) reduction in family stressors; and (7) improved family problem solving and management of challenging behaviors.

#### Research on IHBT

The majority of research studies evaluating impact of intensive home-based interventions have focused on stabilizing families and reducing out of home placements for youth involved in the child welfare and juvenile justice systems (Moffett et al. 2018). For example, Homebuilders (Kinney et al. 1977), a 4–6 weeks “family preservation” program, has been the subject of many controlled studies and reviews. Over time, states and other child-serving systems adapted the Homebuilders model to serve youth involved in mental health (Lindblad-Goldberg et al. 1998; Stroul 1988) and juvenile justice systems (Henggeler 2011). Multisystemic Therapy (MST; Henggeler 2011; MST Services 2020), for example, utilizes an intensive home-based service delivery model to reduce adolescent substance abuse and offending behaviors, and is one of the most extensively researched evidence-based practices for juvenile justice involved youth, with 79 studies and 28 randomized clinical trials.

IHBT programs to reduce risk of out-of-home placement due to psychiatric impairment or SED have proliferated greatly in the U.S. over the past three decades, but controlled research on their effectiveness is less developed. IHBT was cited early in the systems of care movement as a means for addressing gaps in community-based children’s mental health services that led to unnecessary institutionalization, and was the focus of a monograph by one of the co-authors of the original monograph on Systems of Care (Stroul 1988). By 1999, 35 states offered some form of IHBT to children and youth. By 2016, IHBT programs were found to serve hundreds of thousands of children in nearly every state at costs in the tens to hundreds of millions. For example, in FY2018, Virginia expended $145.6 million in federal (Medicaid and Children’s Health Insurance Program) and state funds on IHBT services (Virginia Department of Medical Assistance Services 2018).

However, while child welfare- and juvenile justice-derived IHBT programs have been subjected to dozens of randomized trials, a recent review found only five controlled studies of IHBT for youth with emotional and behavioral impairment (Moffett et al. 2018). Experimental evaluation of a variant of MST called MST-Psychiatric found initial gains in emotional and behavioral functioning that favored the treatment group, but a follow-up study found no maintenance of these gains at 1 year post-treatment (Henggeler et al. 1999; Schoenwald et al. 2000). A second trial was terminated early due to fidelity control and enrollment problems (Rowland et al. 2005).

Controlled research on other IHBT programs, including an intensive home-based cognitive-behavioral treatment (CBT) program in Ontario (Wilmshurst 2002) and a Home-Based Crisis Intervention for children at risk for psychiatric placement (Evans et al. 1997), found null to very small effects. Finally, Barth et al. (2007) used quasi-experimental methods to evaluate an intensive in-home program adapted from MST (Intercept), in use in in 10 U.S. states, compared to comparable youth served via residential treatment. A non-significant trend of better outcomes (OHP, school attendance, avoidance of legal involvement) was found for the IHBT arm, indicating IHBT was at least as effective as residential treatment, as well as less restrictive and expensive (Barth et al. 2007).

Thus, there are a small number of IHBT models that are well-specified and evidence based, but the research base for IHBT programs specifically targeting youths with SED and behaviors relevant to these youth is scant. Other manualized IHBT programs exist for this population (e.g., IICAPS; Woolston et al. 2007), but have not been subjected to rigorous study. Meanwhile, strictly defined and purveyor-controlled evidence-based psychosocial interventions (EBPIs) such as MST are costly and viewed by providers as difficult to implement (Borntrager et al. 2009; Chorpita et al. 2007, 2011). As a result, they have been found to account for a very small percentage of all youth served—as low as one to three percent (Bruns et al. 2016).

For all the above reasons, most IHBT programs operating in state service systems today are “home grown” programs that are less strict with respect to practice parameters, organizational requirements, training and coaching expectations, and fidelity and outcomes monitoring than manualized EBPIs (Hammond and Czyszczon 2014; Moffett et al. 2018). While such flexibility may make such home-grown IHBT programs more likely to be used in “real world” service systems, it may result in lack of clarity around effective practice elements and necessary program factors, resulting in lower quality practice and poorer youth/family outcomes.

Since the early 2000s, there has been a growing literature on the promise of promoting “common elements” of effective treatment (discrete clinical techniques or strategies extrapolated from a larger set of treatment manuals for EBPIs) that may facilitate quality and outcomes while improving flexibility (Chorpita et al. 2005). Research on broad-based applications of common elements to public systems has been encouraging (Daleiden et al. 2006; Chorpita et al. 2017), but has mostly focused on therapists working in outpatient settings, rather than intensive in-home models.

As a relevant example for IHBT, Lee et al. (2014) used methods similar to those of Chorpita et al. (2005) to aggregate and distill information from controlled research studies on interventions to prevent OHP. That study identified *practice* and *program* elements found to be “common” among manualized programs determined by research to be effective for preventing OHP for youth. However, results from this or other research has not been effectively translated into a defined set of program or practice parameters for IHBT.

While evidence-based IHBT models, such as MST, have robust and multiple quality assurance mechanisms (e.g., therapist, supervisory, and organizational measures of adherence), they account for a small proportion of all IHBT delivered. Meanwhile, only a few state IHBT programs (e.g., Massachusetts, Ohio), have developed and deployed measures of adherence with their IHBT service delivery standards. A systematic effort to synthesize both “evidence-based practice” (from studies such as that by Lee and colleagues) and “practice-based evidence” (such as from these states’ local efforts) to develop a cohesive, well-vetted set of program and practice standards for IHBT could hold promise for use in broad-based practice improvement efforts as well as research on IHBT.

### The Current Study

To fill this gap for the public children’s behavioral health field, the research team sought to develop IHBT program and practice standards through a multi-step iterative process. The process we adopted draws from canonical frameworks for quality assessment in health care such as that of Donabedian (1988), which assumes *outcomes* are influenced by both *structure* (e.g., material or human resources, organizational structure) and *process* (what is actually done in providing care). Study methods also are informed by approaches used by entities such as the Agency for Healthcare Research and Quality (AHRQ) Subcommittee on Quality Measures for Children’s Healthcare, which identifies recommended quality measures based on literature reviews, expert input, and a systematic review process focused on both importance and feasibility (Mangione-Smith et al. 2011).

The current project used a comprehensive review of program models and prior literature reviews (e.g., Lee et al. 2014) and expert informant interviews (including representatives of states with quality frameworks) to develop an initial set of standards that were subjected to a version of the *Delphi* process (Linstone and Turoff 1975) process with a range of experts and informants. The goal was to develop two sets of quality standards for IHBT that align with the Donabedian (1988) framework:

1. *Program standards* Conditions at the level of organization, program, or service system (e.g., 24/7 on-call support, access to flexible funding) that describe the structure and resources of the program and that promote program-level quality and fidelity and positive youth/family outcomes.
2. *Practice standards* Distinct activities or techniques that are expected of interventionists or practitioners and proposed to promote positive outcomes (e.g., active listening, crisis plan development, parent behavior management skills training).

Thse current paper provides an overview of literature review and expert interviews followed by a full description of the methods and results of the *Decision Delphi* process that was used to refine and improve an initial set of IHBT standards over two rounds of expert input and feedback.

## Method

### Step 1: Literature Review

Step 1 of the project consisted of a literature review of relevant IHBT models using relevant search terms (e.g., “home based,” “in-home,” “community-based,” “serious emotional disturbance,” “child,” “adolescent,” “psychiatric,” “intensive,” “behavioral”) using PsycINFO ad Web of Science. Unlike efforts by, for example, Lee et al. (2014) and Moffett et al. (2018), this search did not aim to produce a comprehensive review of controlled research studies, but rather surface unique IHBT models for youths with SED that included adequate descriptions of practice and program elements that could serve as the basis for language for initial program and practice standards.

The search was conducted in 2017 and sought articles published between 1995 and 2016. Reviews such as Lee et al. (2014) and Moffett et al. (2018) then provided independent summaries of models for review and specific potential program and practice elements. A summary of relevant IHBT models was compiled from this scan and reviewed by a subset of the authors (authors 4, 5, 6, and 7) for comprehensiveness. Table 1 presents a summary of relevant models the team used as the basis for constructing initial practice and program standards to be submitted for review by experts (step 2) and subsequent *Delphi* process (step 3).

**Table 1:** Interventions included in review of potential intensive home based treatment program and practice elements

Model	Citation(s)
Multisystemic Therapy (MST)	Henggeler et al. (2009)
MST-Psychiatric	Schoenwald et al. (2000)
HOMEBUILDERS	Kinney et al. (1990)
Intensive In-Home Child & Adolescent Psychiatric Services (IICAPS)	Woolston et al. (2007)
Ecosystemic Structural Family Therapy (ESFT)	Lindblad-Goldberg et al. (1998)
Family Centered Treatment (FCT)	Sullivan et al. (2012)
Integrated Co-Occurring Treatment (ICT)	Cleminshaw et al. (2005)
Intensive Home-Based Treatment (IHBT-OH)	Shepler (1991)
Intercept (Youth Villages)	*Youth Villages Intercept Program Model* (n.d.)
Multidimensional Family Therapy (MDFT)	Liddle (2009)
Integrative Family and Systems Treatment (I-FAST)	Fraser et al. (2014)
Solution-focused Brief Therapy	Berg (1994)
Functional Family Therapy (FFT)	Alexander et al. (2013)
Trauma Systems Therapy (TST)	Saxe et al. (2015)
Brief Strategic Family Therapy (BSFT)	Szapocznik et al. (2003)

### Step 2: Expert Interviews

After completion of the literature review (step 1), we conducted a series of phone interviews with N = 16 IHBT, EBPI, and children’s behavioral health experts. Key informants included children’s leads from state behavioral health authorities (n = 5), researchers involved in developing, studying, and/or disseminating child EBPIs (n = 5), representatives from national and state training and consultation centers (n = 3), representatives of managed care organizations (n = 2), and youth and family advocates (n = 1).

An initial set of potential program and practice quality standards was developed and sent to informants in advance of scheduled interview. Each respondent was asked to provide input on (1) the proposed definition of the IHBT *target population* (“Youth with Serious Emotional Disturbance who are at-risk of placement due to a mental health disorder”); (2) the feasibility and appropriateness of using *manualized EBPIs* to provide IHBT in public systems (and specific EBPIs recommended); (3) the feasibility and appropriateness of using *program elements* as a guide or quality assurance mechanism for IHBT (and specific program elements recommended); and (4) the feasibility and appropriateness of using common *practice elements* (and specific program elements recommended). Informants were also asked to provide input on the most effective and feasible overall approach states and other purchasers might take to assure IHBT quality and any other recommendations. Because results of interviews were used primarily in preparation for step 3 (*Delphi* process), we only provide a brief summary of findings here.

Regarding the definition of the target population, many informants observed that eligibility only for youth with SED risked being too narrow, given many effective in-home programs were built for and could benefit other populations. However, many of these same informants also agreed that funding rules required specific eligibility criteria, and that to be useful, the current project also needed to be clear on the population of focus.

Although many informants believed manualized EBPIs provide a high standard of care, the majority voiced that basing IHBT services provided in routine practice and public systems on one or more EBPIs was unlikely to be feasible or acceptable to most purchasers and providers. Many informants reported that a quality framework based on common elements was likely to be a more feasible, acceptable, and flexible way of using available evidence. As one informant put it, “train and supervise them to do things that work and measure outcomes.” Another voiced, “having some ‘off-the-shelfs’ is powerful, but [states] also need to also build their own [model] for the rest of the population.” A third said, “[EBPIs] are helpful, but also cost more, and don’t necessarily help everyone.” Other common themes focused on the need for infrastructure to aid IHBT providers, such as training and coaching for IHBT practitioners; outcomes and quality monitoring; state centers of excellence that could conduct these activities; and high-quality, frequent supervision. Although several experts noted the influence of such “outer context” (policy and financing) factors, for the purposes of the current project, none proposed a different organizational structure than focusing on the practice and organization domains.

In sum, experts reported that quality indicators or implementation standards, aligned with research-based program and practice elements, was likely to be an important route to more focused quality improvement by states and other purchasers. As such, the results supported proceeding to Step 3. Informants also provided feedback on preliminary indicators of quality at the practice and program levels submitted for their review and nominated additional potential indicators of high-quality and/or research-based IHBT. This information aided in finalizing a first set of items for review and a participant list for the *Delphi* process.

### Step 3: Delphi Process

Experts in the field of IHBT were recruited to participate in a web-based *Delphi* process (Linstone and Turoff 1975). The *Delphi* process is based on the principle that decisions from a group of informed individuals will be more accurate than from a small number of people or an unstructured group. *Delphi* employs a structured method through which a panel of experts answer questionnaires in two or more rounds. After each round, the facilitator reports back results from the previous round with detailed feedback from those who rendered judgments. In subsequent rounds, experts are thus encouraged to revise earlier ratings or answers in light of results from the panel overall. The process is stopped after a predefined number of rounds or when a criterion is reached based on stability of results. The Delphi process as used here adhered closely to hallmarks of a standard Delphi process, in that it relied on an external facilitator or “change agent,” employed a heterogeneous group of participants (who were nonetheless informed on the topic), allowed participants to review anonymous feedback from other participants, and sought to use no fewer than two rounds of feedback to achieve a high level of agreement (Linstone and Turoff 2002).

For the current project, experts again included state children’s mental health directors from the National Association of State Mental Health Program Directors (NASMHPD), developers of evidence-based practice models, major providers of IHBT across the country, parent and youth leaders with perspectives on IHBT, and representatives of national and state children’s mental health technical assistance centers. Initially invited participants were also asked to identify, through a snowball process, further stakeholders with expertise in IHBT who should engage in the Delphi process.

#### Delphi Process Survey

Respondents were asked to rate each standard’s (1) importance and feasibility with respect to fundamental *content*, and (2) clarity and appropriateness of proposed *wording.* For *content*, each participant indicated whether the standard described was *essential, optional, or inadvisable* for IHBT programs. They were also asked to provide their rationale, concerns, or other information related to the standard’s capacity to promote positive outcomes (or, in the case of program standards, effective practice and positive outcomes). For *wording,* each participant indicated whether, as written, the description of the activity was *acceptable*, *acceptable with minor revisions*, or *unacceptable*. For wording, *Delphi* participants had the opportunity to propose alternative language if they viewed an item *acceptable with minor revisions* or *unacceptable* as written. A financial incentive of $30 for review of each set of standards in each round was provided.

#### Criteria for Acceptance

The research team applied a set of criteria for standards to be approved. To meet criteria for “Full Approval,” 75% or more of respondents had to rate the standard’s *content* as “Essential” or “Optional,” and 75% or more had to rate the *wording* as “Acceptable.” A standard was considered to have met criteria for “Partial Approval” if it met one of the above criteria, but not both. Standards rated as “Inadvisable” for content by > 50% of respondents were removed from further consideration.

For standards that did not meet the above criteria for “Full Approval,” qualitative data were coded using standard techniques (e.g., Auerbach and Silverstein 2003) to identify unique pieces of feedback about the content or wording of standard across all Delphi participants. These codes were then sorted into themes, defined as “broad units of information that consist of several codes aggregated to form a common idea” (Creswell 2013, p. 186). A summary of qualitative analyses of open-ended input (all themes/categories and N of respondents who contributed a comment coded into that theme) was fed back to *Delphi* participants after each round of review, as per recommendations for effective use of the *Delphi* process*.*

## Results

Because literature review and expert interviews were primarily used to inform the development of items to be reviewed by experts in the *Delphi* process (step 3), we have briefly reported the results of steps 1 and 2 above in the “Methods” section. Results reported below thus focus only on results of the *Delphi* process.

### Delphi Participants

A total of 150 informants were invited to review and rate the program and practice standards. Twelve invited respondents (8%) opted out due to self-reported lack of content expertise. Of the remainder, a total of 74 invitees returned complete practice standards surveys (54%) in round 1, while 58 (42%) returned complete surveys of program standards. For round 2, we sent revised standards for both program and practice to all 74 participants who provided feedback on at least one set of standards in round 1. Thirty-eight participants (51%) returned complete surveys in round 2 for both program and practice standards. To analyze the possibility of differential attrition, independent samples t-tests were used to evaluate differences in round 2 survey completers and non-completers, using Bonferroni corrections, for round 1 scores on both practice standard ratings (p < .001) and program standard ratings (p < .001). No significant differences were found between respondents who provided one versus two rounds of ratings.

#### Characteristics of Respondents

Table 2 presents a full summary of *Delphi* respondents. As shown, the most commonly reported roles were state service system administrators (50%) and consultants/technical assistance providers (22%). The mean number of years of experience in children’s behavioral health was 23. The majority of respondents were white (86%) and reported having a Master’s level of education or higher (95%), primarily in social work, clinical psychology and counseling. As was the case for differences in round 1 ratings, we found no significant differences between reviewers who participated in one versus both rounds of feedback by role, years of experience, or any other characteristics measured.

**Table 2:** Characteristics of Delphi participants

Variable	Practice standards (N = 74)		Program standards (N = 58)	
	N	Valid percent	N	Valid percent
Role				
State service system administrator	37	50.0	29	50.0
Local service system administrator	3	4.1	2	3.4
Direct service provider	1	1.4	1	1.7
Administrator or manager in a provider organization	4	5.4	4	6.9
Youth advocate	1	1.4	1	1.7
Family advocate	1	1.4	1	1.7
EBP developer/purveyor	3	4.1	3	5.2
Consultant/technical assistance provider	16	21.6	13	22.4
Other	8	10.8	4	6.9
Region				
West	5	10.9	3	8.1
Southwest	1	2.2	2	5.4
Midwest	9	19.6	8	21.6
Northeast	12	26.1	9	24.3
Southeast	4	8.7	5	13.5
National/not reported	15	32.6	10	27.0
Education				
Bachelor’s degree	2	2.7	2	3.5
Master’s degree	54	74.0	39	68.4
Doctoral degree or equivalent	16	21.9	15	26.3
Other (Medical Assistant, RN, etc.)	1	1.4	1	1.8
Primary language				
English	71	97.3	55	96.5
Spanish	2	2.7	1	1.8
Other	0	.0	1	1.8
Gender identity				
Male	21	28.4	19	33.3
Female	53	71.6	38	66.7
Non-binary/other	0	0	0	.0
Race/ethnicity				
African American	5	6.8	2	3.4
Native American/Alaska Native	0	0	0	0
Hispanic/Latino	4	5.4	4	6.9
Asian American	3	4.1	1	1.7
White/Caucasian	62	83.8	51	87.9
Other	1	1.4	1	1.7

Participant characteristics shown are for respondents in Round 1. Only respondents who completed Round 1 were invited to participate in Round 2

### Delphi Results

#### Program Standards, Round 1

**Fig. 1:**
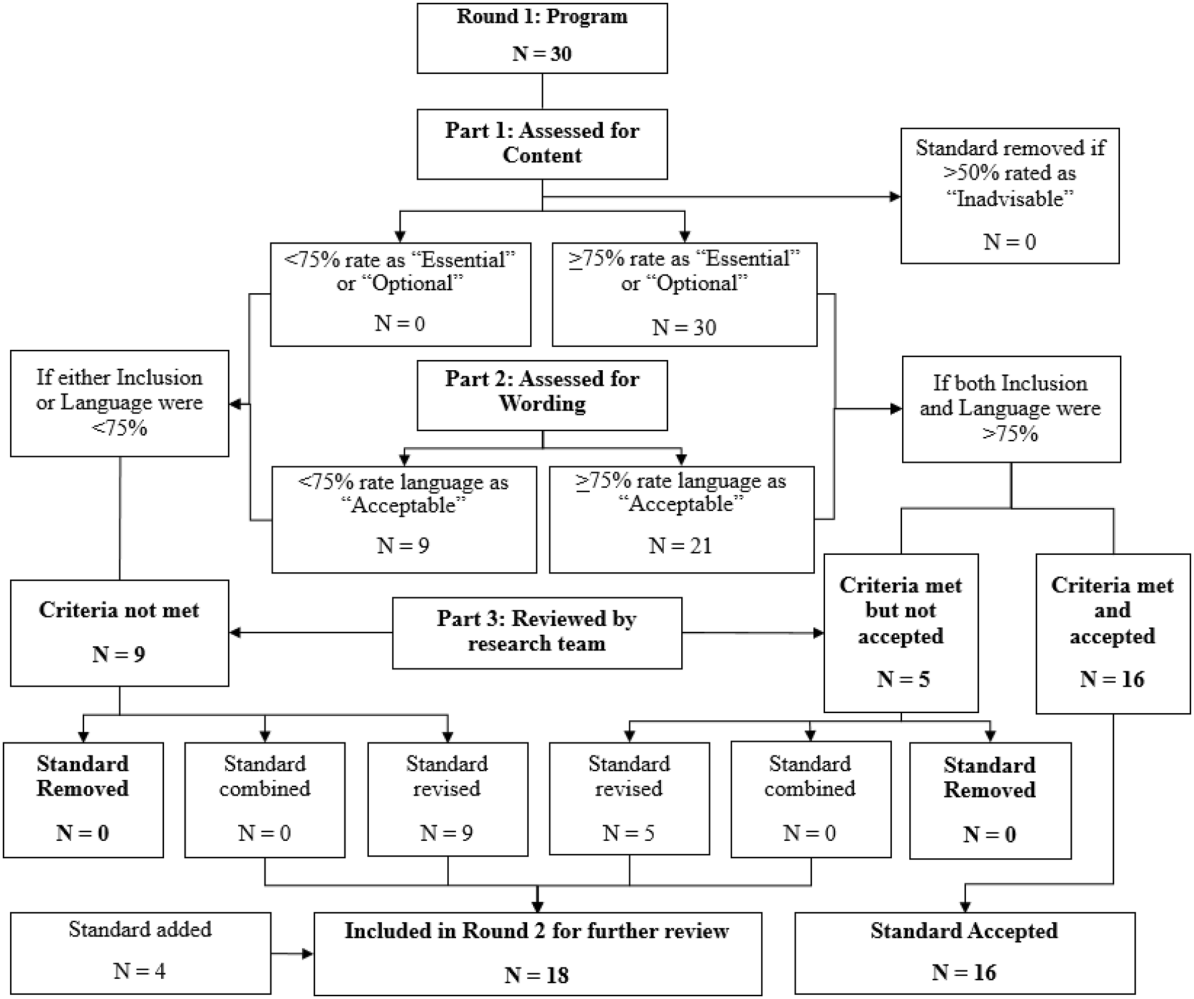
Delphi process flow chart for program standards (round 1)

**Fig. 2:**
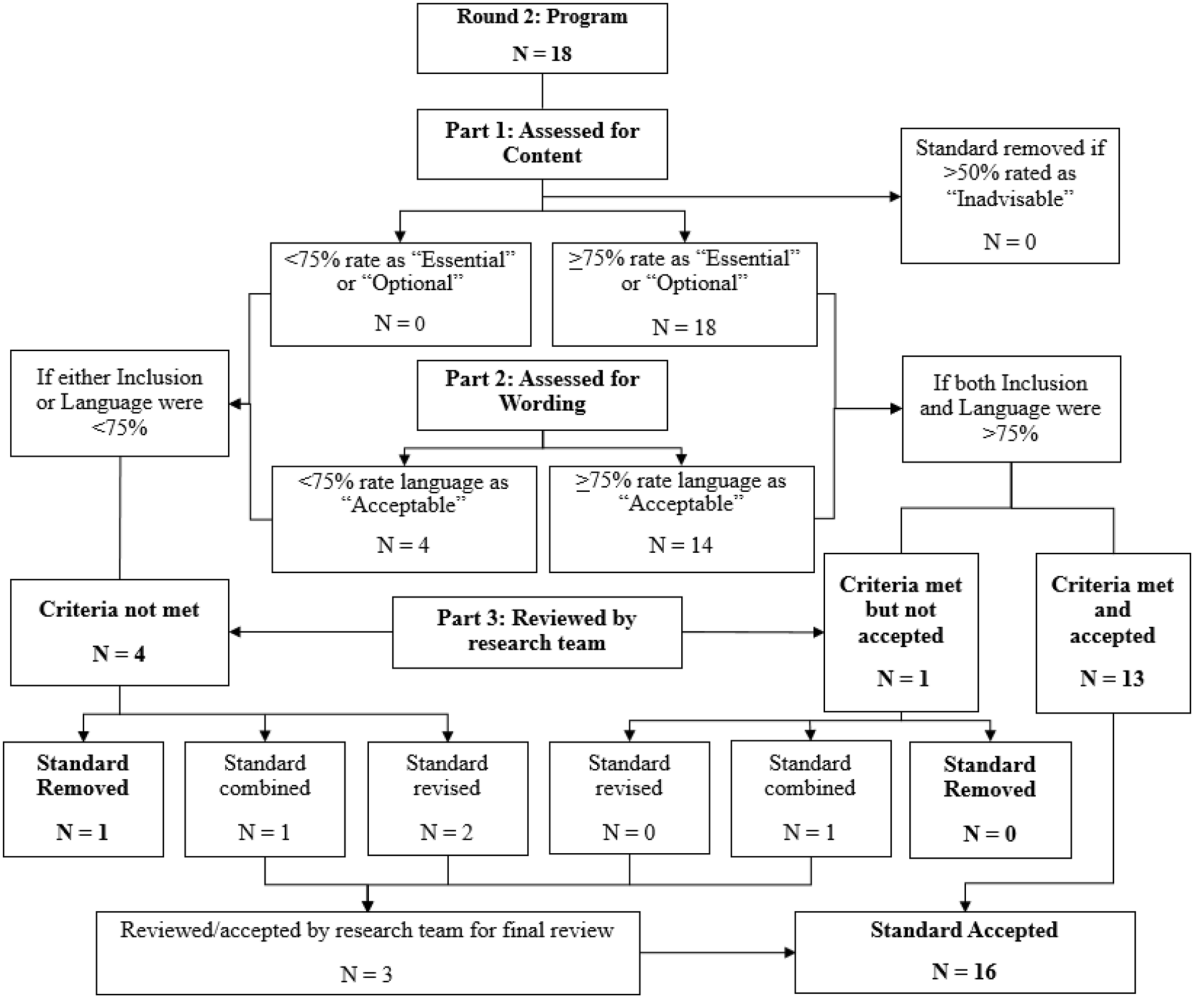
Delphi process flow chart for program standards (round 2)

Figures 1 and 2 present the sequence of review and revision steps and ultimate decisions for *program standards* across Rounds 1 and 2, respectively. As shown, 30 program standards were submitted to respondents for review in Round 1. After Round 1 of the Delphi process, 21 standards met criteria for approval (both content and wording were > 75%), and 9 standards met criteria for partial approval (one but not both of these criteria met). The research team revised the 9 standards that received partial approval based on qualitative feedback from Delphi respondents, as well as 5 standards that the research team felt could be improved based on feedback, despite meeting criteria for approval. For a summary of all program standards reviewed, the percent of Delphi participants who approved both content and wording, and the disposition of each standard across rounds 1 and 2, see Table 3.

**Table 3:** Percent of proposed program standards approved for content and language, Delphi rounds 1 and 2

	Approval rating^a^						
	Round 1 (N = 58)			Round 2 (N = 38)			
	Content^b^	Language^c^	Result^d^	Content^b^	Language^c^	Result^d^	Program Standard^e^
1. Role clarity	100.0	89.7	A	–	–	–	1.1
2. Practitioner credentials	100.0	77.6	A	–	–	–	1.2
3. Qualified personnel	96.6	74.1	E	97.4	71.1	C	1.3
4. Stable workforce	96.6	64.3	E	92.1	75.7	A	1.4
5. Rigorous hiring processes	98.3	75.0	A/E	89.4	76.3	A	1.5
5B. Reflective hiring process			N	97.3	83.8	A	1.6
6. Effective training	100.0	78.6	A	–	–	–	1.7
7. Initial apprenticeship	94.8	71.9	E	100.0	80.6	A/C	1.3
8. Ongoing skills-based coaching	100.0	91.4	A	–	–	–	1.8
9. Intensive supervision	94.8	76.4	A/E	97.4	78.9	A	1.9
10. Quality of supervision	98.3	75.4	A/E	97.4	86.5	A	1.10
10B. On call support			N	97.3	81.6	A	1.11
11. Clear eligibility criteria	100.0	77.6	A	–	–	–	2.1
12. Practice protocols	100.0	79.0	A/E	94.3	83.3	A	2.2
13. Service coordination	100.0	66.1	E	94.6	83.8	A	2.3
13B. Lead Clinical Role			N	81.1	62.9	R	–
14. 24/7 availability	100.0	82.5	A	–	–	–	2.4
15. Commitment to flexibility and accessibility	100.0	91.1	A	–	–	–	2.5
16. Ecological focus	100.5	83.9	A	–	–	–	2.6
17.Comprehensiven-ess of intervention	100.0	71.9	E	97.2	69.4	E	2.7
18. Safety planning	100.0	66.7	E	100	94.4	A	2.8
19. Small caseloads	100.0	73.2	E	100	81.1	A	2.9
20. Intensity of Intervention	100.0	61.4	E	100	75.0	A	2.10
21. Focused treatment duration	100.0	75.9	A/E	97.3	86.5	A	2.11
21B. Post transition services			N	89.2	63.9	E	2.12
22. Outcome monitoring	100.0	83.9	A	–	–	–	3.1
23. Quality monitoring	100.0	89.5	A	–	–	–	3.2
24. Effective data management	100.0	84.2	A	–	–	–	3.3
25. Review of care plans	100.0	63.6	E	94.7	89.2	A	3.4
26. Comprehensive system collaboration	100.0	79.0	A	–	–	–	4.1
27. Positive work environment	100.0	85.7	A	–	–	–	4.2
28. Effective leadership	100.0	82.5	A	–	–	–	4.3
29. Adequate compensation	100.0	92.7	A	–	–	–	5.1
30. Routine oversight of key operations	100.0	86.0	A	–	–	–	5.2

^a^Not all respondents rated each standard

^b^Approval rating for “content” = percent of respondents who rated a standard as “Essential” or “Optional”

^c^Approval rating for language = percent of respondents who rated the wording of a standard as “Acceptable”

^d^Result column denotes the result of the round: “A” = standard met criteria; “E” = standard edited; “R” = standard removed; “C” = standard combined with another standard; “N” = new standard added

^e^Standard column indicates the number ultimately assigned in the Program Standards document

#### Example of Revision

An example of the feedback and editing process is provided by Program standard 25, which received partial approval. Although the majority of respondents agreed the standard should be included, approximately half objected to the original language:

Review of care plans: Each youth /family’s initial plan of care is reviewed by an expert in the IHBT practice model (ideally external to the supervisor or coach). Updated plans of care should be reviewed no less than bi-monthly.

Thematic coding of the qualitative responses revealed to two major concerns: (1) requiring an external reviewer would cause an undue burden on programs since they may not have access to or funds available for such an effort and that a supervisor should therefore be adequate to fill this role; and (2) specifying a bi-monthly review was too prescriptive. This feedback was taken into account and revisions made, resulting in the following revised standard:

Review of care plans: Each youth and caregiver's initial plan of care is reviewed by an expert (e.g., a supervisor) in the IHBT practice model. Updated plans of care should also be regularly reviewed.

Based on input, the research team also created 4 new standards during the review phase of Round 1 (e.g., based on input about missing concepts that needed to be added, or multi-barreled standards that reviewers suggested should be the basis for two unique standards). Thus, after Round 1, 16 standards were formally accepted, and 18 standards proceeded to a second round of review and comment.

#### Program Standards, Round 2

After review by respondents in Round 2, 14 remaining standards met criteria for full approval, and 4 received partial approval. One of the partially approved standards, “IHBT practitioner serves a lead role,” was removed, due to multiple objections from reviewers that in complex public service systems with many other services and initiatives, system processes may dictate that another entity serve in a lead role (e.g., a care coordination unit). Another partially approved standard, “Qualified personnel,” was combined with an approved standard, “Initial apprenticeship,” to reduce redundancy.

The remaining 2 partially approved standards narrowly missed the cut-off for outright approval. Specifically, “Comprehensiveness of intervention” and “Post transition services,” both met criteria for content (97.2% and 89.2% respectively), but fell short of the 75% threshold for language (69.4% and 63.9%). The research team made minor revisions to the language of these two standards, thus bringing the total to 16 approved standards in Round 2. Thus, after two rounds consensus among experts was established, resulting in 32 final program standards.

#### Practice Standards, Round 1

**Fig. 3:**
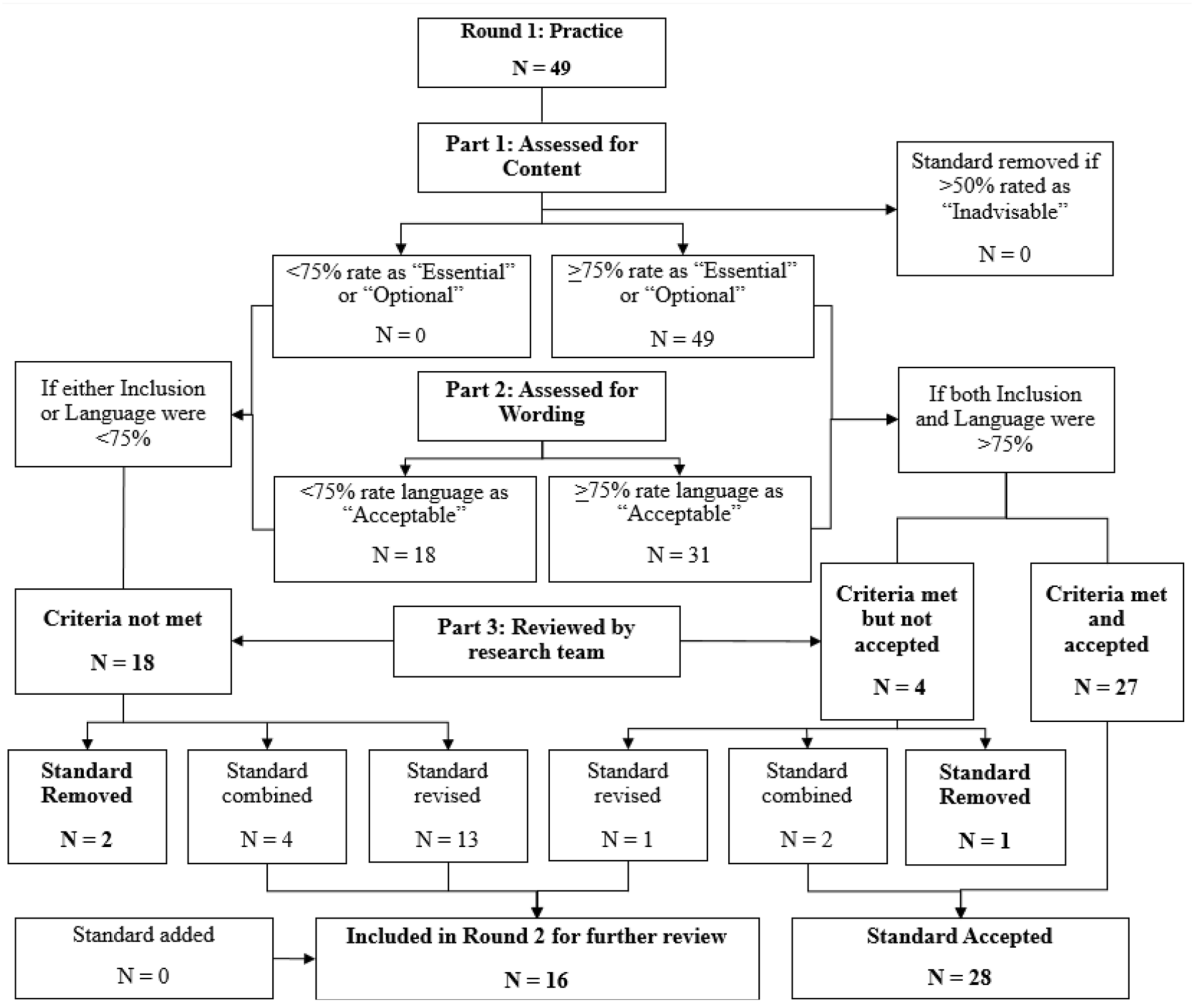
Delphi process flow chart for practice standards (round 1)

**Fig. 4:**
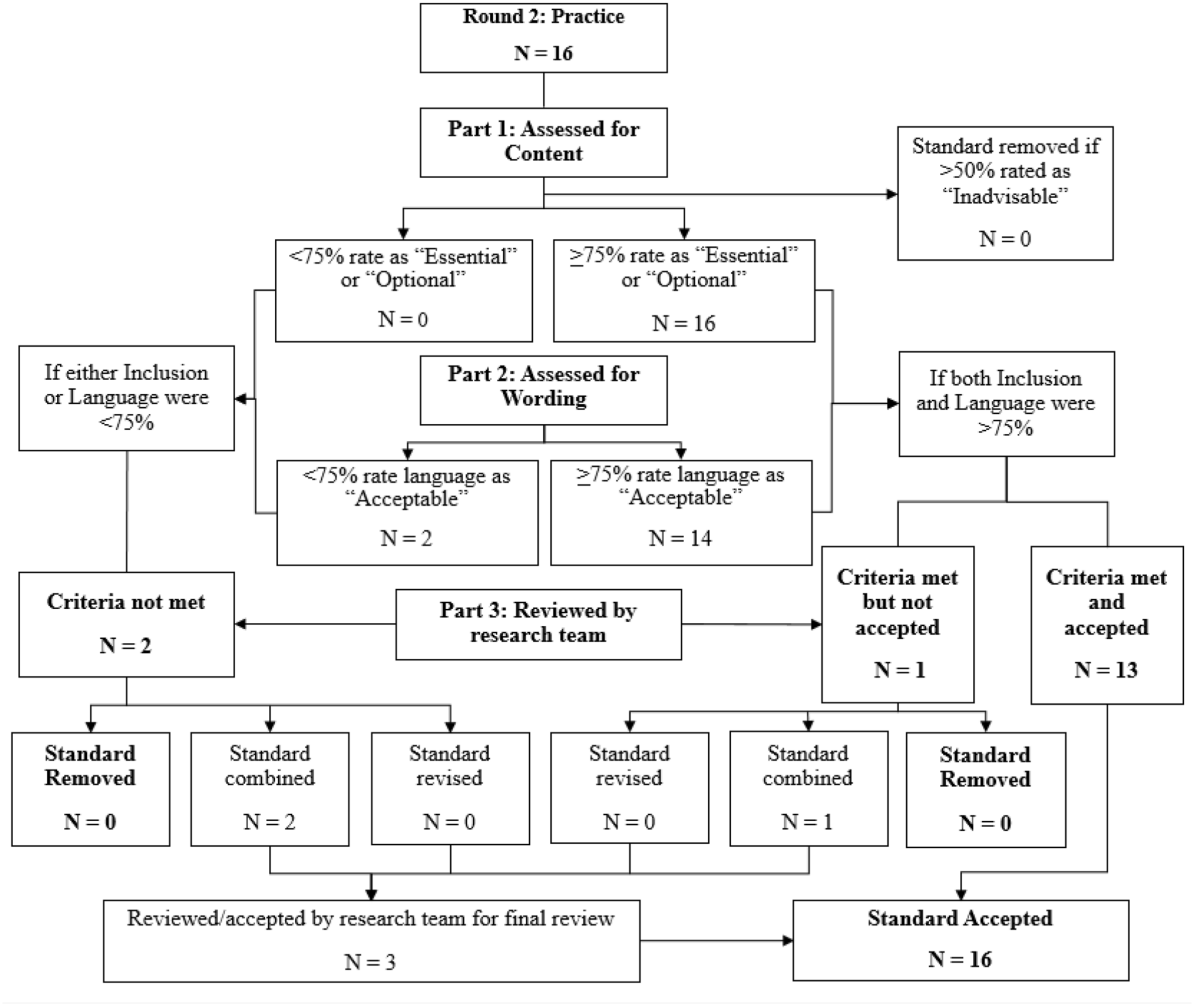
Delphi process flow chart for practice standards (round 2)

Figures 3 and 4 present the sequence of review and revision steps and ultimate decisions for practice standards across Rounds 1 and 2, respectively. As shown, 49 practice standards were submitted to respondents for review in Round 1. After Round 1 of the Delphi process, 31 standards met criteria for approval (both content and wording were > 75%), and 18 standards met criteria for partial approval. Of the 18 standards receiving partial approval, 13 standards were revised, 4 standards were combined (becoming 2 standards), and 1 standard was removed. Of the 31 fully approved standards, reviewer feedback led to revision of one standard and removal of one standard. Two standards, “Identifies additional supports needed” and “Builds family resources and supports,” were combined into one approved standard. Thus, after Round 1, 28 standards were accepted, and 16 standards proceeded to a second round of review. For a summary of all practice standards reviewed, the percent of Delphi participants who approved both content and wording, and the disposition of each standard across rounds 1 and 2, see Table 4.

**Table 4:** Percent of proposed practice standards approved for content and language, Delphi rounds 1 and 2

	Approval rating^a^						Practice
	Round 1 (N = 74)			Round 2 (N = 38)			
	Content^b^	Language^c^	Result^d^	Content^b^	Language^c^	Result^d^	Standard^e^
1. Describes IHBT	100.0	73.0	E	100	92.1	A	1.1
2. Explains confidentiality	100.0	87.8	A	–	–	–	1.2
3. Engages the youth/caregivers/family	100.0	88.5	A	–	–	–	1.3
4. Employs motivational enhancement strategies	95.9	73.6	E	97.4	89.5	A	1.4
5. Actively seeks to understand	100.0	73.2	E	100	94.7	A	2.1
6. Uses language that is accessible	91.9	62.5	E	97.3	86.8	A	2.2
7. Identifies risks	98.6	69.4	E	100	84.2	A	3.1
8. Co-creates safety plan	100.0	51.4	E	100	81.6	A	4.1
9. Regularly monitors and updates the safety plan	100.0	77.5	A	–	–	–	4.2
10. Responds to crises	95.8	69.4	E	100	83.8	A	5.1
11. Uses crisis de-escalation	100.0	94.4	A	–	–	–	5.2
12. Identifies needs and current functioning	100.0	87.5	A	–	–	–	6.1
13. Identifies functional strengths	100.0	82.9	A	–	–	–	6.2
14. Assesses for trauma	98.6	63.0	E	100	94.4	A	6.3
15. Creates functional understanding of behavior	100.0	76.4	A	–	–	C	7.2
16. Prioritize needs	100.0	66.7	E	97.4	81.6	A	7.1
17. Conducts a function analysis	98.6	66.7	E	94.7	73.0	C	7.2
18. Develops definition of needs and goals	98.6	63.9	R	–	–	–	–
19. Develops treatment goals	98.6	71.6	C	97.4	92.1	A	8.1
20. Develops treatment plan	100.0	64.7	C	97.4	92.1	A	8.1
21. Develops indicators of progress	100.0	84.3	A	–	–	–	8.2
22. Provides psychoeducation	98.6	76.1	A	–	–	–	9.1
23. Conducts standardized assessment	100.0	80.3	A	–	–	C	10.1
24. Develops individualized indicators	98.6	72.2	E	94.7	73.7	C	10.1
25. Builds youth skills	100.0	78.6	A	–	–	–	11.1
26. Builds caregiver skills	97.3	71.2	E	100	76.3	A	12.1
27. Promotes positive relationships	100.0	76.4	A	–	–	–	12.2
28. Uses cognitive-behavioral strategies	100.0	83.3	A	–	–	–	13.1
29. Promotes positive family interactions	100.0	81.7	A	–	–	–	14.1
30. Ensures collaboration	97.3	68.1	C	100	81.6	A	15.1
31. Leads collaboration	98.6	63.9	C	100	81.6	A	15.1
32. Assesses for substance abuse treatment needs	98.6	77.5	A	–	–	–	15.2
33. Assesses for social service needs	100.0	75.7	A	–	–	–	15.3
34. Promotes positive relations with systems	98.6	78.9	A	–	–	–	16.1
35. Arranges for supports from systems	98.6	80.3	A	–	–	C	16.2
36. Provides system navigation	97.3	70.0	E	100	92.1	A	17.1
37. Develops accommodations	98.6	77.9	A/E	97.4	84.2	A/C	16.2
38. Builds community assets	100.0	87.1	A	–	–	–	18.1
39. Builds a future orientation	98.6	81.7	A	–	–	–	18.2
40. Identifies current family resources and supports	97.3	75.0	A	–	–	–	19.1
41. Identifies additional supports needed	100.0	76.1	A/C	–	–	–	19.2
42. Builds family resources and support	97.2	76.8	A/C	–	–	–	19.2
43. Establishes transition criteria	100.0	82.6	A	–	–	–	20.1
44. Develops post-IHBT crisis plan	100.0	88.7	A	–	–	–	20.2
45. Develops skill maintenance plan	100.0	85.7	A	–	–	–	20.3
46. Provides linkage to post-IHBT resources	100.0	82.4	A	–	–	–	20.4
47. Discusses access to future IHBT services	100.0	88.4	A	–	–	–	20.5
48. Schedules closing session	100.0	90.0	A	–	–	–	21.1
49. Creates check-in procedure	94.4	76.8	A/R	–	–	–	–

^a^Not all respondents rated each standard

^b^Approval rating for “content” = percent of respondents who rated a standard as “Essential” or “Optional.”

^c^Approval rating for language = percent of respondents who rated the wording of a standard as “Acceptable.”

^d^Result column denotes the result of the round: “A” = standard met criteria; “E” = standard edited; “R” = standard removed; “C” = standard combined with another standard

^e^Standard column indicates the number ultimately assigned in the Program Standards document

#### Practice Standards, Round 2

After review by respondents in Round 2, 14 remaining standards met criteria for full approval, and 2 received partial approval. One partially approved standard, “Conducts a functional analysis,” was combined with “Creates functional understanding of behavior,” which was approved in round 1. Similarly, “Develops individualized indicators of progress,” was combined with “Conducts standardized assessment,” also approved in round 1. Additionally, one approved standard, “Develops accommodations” was combined with a standard approved in Round 1, “Arranges for supports from systems.” Thus, 13 new standards were accepted and 3 standards were combined with previously approved standards. After two rounds of the Delphi process, consensus amongst experts was established, resulting in 41 final practice standards.

## Discussion

Public behavioral health systems in the U.S. expend hundreds of millions of dollars on IHBT with an aim of improving outcomes for children and youth with SED and their families and reduce reliance on institutional care. As implemented in the real world, however, the quality of these services is variable. Although an array of intensive home-based EBPIs exist, their effectiveness for youths with SED is not well-established, and those that may be relevant are rarely used in public systems. Alternative approaches are needed to assure that IHBT implementation is guided by systems of continuous quality improvement (CQI). At a minimum, such systems should operationalize and continuously measure three things: program structures, practices provided, and client outcomes (Donabedian 1988).

### Overview of Findings

An initial set of IHBT quality standards was positively viewed by a diverse group of experts and stakeholders, with 70% of program standards and 63% of practice standards meeting criteria for acceptability after only one round of rating and input. After revision and a second round of input, 88% of proposed program standards and 95% of practice standards reached criteria. After a last round of minor revisions, a set of 32 program and 48 practice standards was finalized and disseminated to what had evolved into a national learning community of IHBT experts and stakeholders (see Appendix Tables 5 and 6).

The achievement of relatively high ratings of importance, acceptability, and feasibility by experts over just two rounds of feedback was facilitated by a number of factors, including prior research on common elements of effective community-based interventions for youth with SED (e.g., Lee et al. 2014), nomination by like-minded peers, and alignment of the standards with common wisdom among experts and stakeholders. Prior development of quality standards for other community children’s mental health models that had evolved out of “practice-based evidence,” such as Treatment Foster Care (Farmer et al. 2002), Wraparound (Walker and Bruns 2006), and parent peer support (Olin et al. 2015) also provided a precedent for the development of the IHBT standards that increased the credibility of the process and its results. Finally, the increasing awareness of the importance of quality metrics in children’s behavioral healthcare, and the presence of well-accepted methods for developing them (e.g., Mangione-Smith et al. 2011), aided the process.

### Themes from Expert Consensus Process

For many standards, stakeholders from state behavioral health authorities and provider agencies voiced that quality indicators should be more feasible than initially presented. For example, many EBPIs reviewed in step 1 of the project mandate review of treatment plans by an external expert or coach, a step viewed as infeasible by many state representatives and providers. After editing to accommodate feedback from stakeholders, the final, revised program standard (No. 25: “Review of care plans,” see Appendix Table 5) states that “Each youth and caregiver’s initial plan of care is reviewed *by an expert* in the IIHBT practice model,” without specifying that the expert must be external to the provider organization. This is but one of many examples where the core team balanced feedback from experts who advocated for a “gold standard,” often based in expectations for established EBPIs, against the need for feasibility. By feeding back both quantitative and qualitative input, the *Delphi* process allowed participants to consider their own ratings and perspectives against that of their peers. The high level of endorsements of revised standards (round 2) suggests that the process facilitated achievement of an appropriate balance.

A second theme in the development, review, vetting, and revision of the standards was issues related to the overlap of functions, responsibilities, and services in public systems of care. For example, many intensive in-home EBPIs, such as MST, require the therapist to manage all aspects of the youth and family’s treatment. However, in many systems and/or for certain populations, this function may be performed by other providers, such as a Wraparound care coordinator. Similar questions arise around the IHBT program’s ability to be the first crisis response option for a family, such as when a mobile response and stabilization service (MRSS; Manley et al. 2018) exists in the system of care. When designing local continuums of care, it is important to clearly distinguish the discrete, as well as, overlapping roles and functions of IHBT in relation to programming such as Wraparound and MRSS, including decision protocols for determining which service has the primary responsibility for overlapping functions.

Finally, application of IHBT practice standards must account for the wide range of experience and training that is represented in the IHBT workforce. Despite the complexity of the work, many IHBT practitioners are new graduates of Masters or even Bachelors programs, with little experience, while others may be seasoned veterans and be certified in one or more specific EBPIs.

In developing and revising standards based on feedback, the research team adopted a mindset that *program standards* should be consistent expectations of states and other purchasers of service, written into contracts and memoranda of agreement and designed to enhance the consistency of implementation of IHBT model components. Particularly because many IHBT practitioners are new to the field, intensive supervision and ongoing training are among these expectations of IHBT programs. *Practice standards,* however, might better be viewed as competency targets, designed to highlight key practitioner skills and competencies that are necessary for quality delivery of IHBT. As such they become a focus for the development and enhancement of clinical competencies through ongoing training, intensive supervision, and clinical consultation. One would expect that more experienced, well-supported IHBT practitioners will meet the majority of these standards, while novice practitioners will require more time to do so.

### Application of the IHBT Standards

It is our hope that the IHBT standards developed through this project can inform future efforts to improve the quality of IHBT programs and outcomes experienced by youth and families. Encouragingly, immediately after completion of this study, a national IHBT learning community of states, managed care entities, and large behavioral health provider organizations was launched that allows participants to gain insights from peers about how the standards could promote quality assurance and inform contracting, financing strategies, and investments in workforce development.

In the future, we envision translating the IHBT standards into measurement strategies that are employed in more rigorous approaches, such as *quality collaboratives* (ØVretveit et al. 2002) that use data on relevant metrics to identify change targets, inform improvement plans, and evaluate success or progress. Quality improvement efforts using IHBT standards could also include value-based payment (VBP) arrangements that provide additional reimbursement to providers for meeting quality and outcome benchmarks (Institute of Medicine 2007), thus serving the dual purpose of enhancing the quality of IHBT service delivery and providing additional resources to the program to offset the additional costs of providing IHBT to fidelity. Such VBP efforts could address feasibility concerns of the standards (discussed above), by providing resources needed by providers to meet standards, rather than adjusting research-based practices further due to feasibility concerns.

Finally, standards such as those developed for IHBT can link to broader federal quality initiatives. In the child welfare sector, these standards can aid in development and evaluation of outcomes (e.g., preventing or reunifying children after out-of-home placement) for programs included in state plans under the Family First Prevention Services Act (FFPSA; Lindell et al. 2020). In healthcare, among the many provisions to strengthen care quality, the Child Health Insurance Program Reauthorization Act (CHIPRA) requires states to report on a core set of health care quality measures by 2024. Although standards such as these for IHBT extend far beyond the CHIPRA Core Set (which are still under development), federal quality initiatives may increase willingness and capacity to pursue other metric-based quality improvement efforts. Moreover, availability of IHBT—and adherence to research-based standards—hold promise for improving states’ status on specific measures in the Core Set, such as follow-up after hospitalization for mental illness or appropriate medication prescribing and monitoring.

### Limitations and Need for Future Research

A typical limitation of efforts to define quality metrics or standards is that they are not fully based on rigorous research on what factors are associated with client outcomes. To bridge this gap in knowledge, and to accommodate the need to assure feasibility and acceptability, expert and stakeholder opinion is used as a key input to the process (Mangione-Smith et al. 2011). In the example of the current project, the literature on effective IHBT programs for youth with SED is particularly scant, limiting the degree to which a comprehensive, formal meta-analysis on interventions for the target population could even be conducted.

Although this represents a limitation of the current project, efforts are now under way to develop quality measures based on these indicators which can then be used in future research. For example, the relationship between standards adherence and outcomes should be evaluated, in order to establish the validity of these measures and expand the research base on IHBT. This has been done previously for other models that were based on “practice-based evidence” in the children’s behavioral health field. For example, Farmer et al. (2002) developed quality measures for Treatment Foster Care based on newly established standards, and found scores from these measures were associated youth outcomes. For other intensive models, including Wraparound (Bruns et al. 2008) and Assertive Community Treatment (Salyers et al. 2003), a combination of norm-referencing (i.e., review of score distribution for many programs) and criterion-referencing (i.e., association with outcomes) have been used to establish quality benchmarks for fidelity measures. Such research will be needed to inform the field on which standards are essential versus not essential, appropriate uses of the standards, and the types of decisions that should—and should not—be made on the basis of the scores.

A second set of limitations relate to the specific methods that were chosen for the current study. For example, we chose to define program and practice domains based on past research and expert interviews. The results may have differed if the domains and initial standards were defined by experts themselves via methods such as Concept Mapping (Trochim 1989). Additionally, we did not evaluate and adjust for distinct response patterns across participants (or types of participants), which could have been evaluated using measurement models such as those based on item response theory (IRT; Hambleton et al. 1991). The above approaches could be used in the future to validate or supplement the IHBT standards.

A third set of limitations relate to the characteristics and attrition of Delphi process participants. First, although nearly half of experts engaged in initial interviews were EBPI developers and purveyors, the majority of Delphi participants were representatives of public service systems. This was the result of inviting all state children’s behavioral health leads to participate, in an effort to achieve external validity. However, this imbalance may have influenced results. There also was attrition from round 1 to round 2 of the Delphi process, whereby loss of raters for program standards was 34%, and practice standards was 47%. Although no significant differences were found between experts completing one survey versus both surveys (on either respondent characteristics or round 1 scores), it is possible some unmeasured factor differentiated these groups, yielding biased results in round 2. It is also not clear why respondents were less likely to complete the second round of practice standards review, although the greater number of practice standards may have been a factor.

Finally, although consensus was not perfect after the second round of input, we chose not to conduct a third round of the Delphi process. This decision was based on the near-complete consensus that was achieved after round 2 (only 4 of 34 program standards and 2 of 44 practice standards did not reach criteria). Nonetheless, it must be acknowledged that experts and stakeholders did not formally review the small number of final revisions.

## Conclusion

In conducting this study, we were reminded repeatedly of the many commonly cited limitations of public systems, including funding and workforce limitations that may constrain achievement of these standards. We were also reminded of how rapidly and dramatically the context of behavioral healthcare provision can change. Since completion of this study, the COVID-19 (SARS Co-V-2) pandemic has forced modifications to IHBT practice and illuminated disparities in healthcare, and a national racial justice movement has brought further attention to the need to address disparities in healthcare access and outcomes. Such events point to potential shortcomings in the IHBT standards related to structural racism and social determinants of health.

For example, in addition to standards related to identifying youth and family strengths, needs, and resources, a standard could be included in the “comprehensive contextual assessment” section of the practice standards that explicitly demands attention to collecting information on social determinants of health (e.g., safe housing, transportation, safety of neighborhood; racism, discrimination, and violence; education, job opportunities, and income). Similarly, program standards could call out the need for training and supervision on using such an ecological focus and incorporating anti-racist principles into provision of services. In the Accountability section, a standard could be included related to collecting and disaggregating data on families served and providers that include race, ethnicity, and language.

Such events only reinforce the need for continually refining the standards based on research as well as feedback from diverse members of the IHBT community. As the standards are applied to CQI and workforce efforts in large-scale IHBT programs and initiatives nationally, feedback will continue to be compiled. Where appropriate, we expect that further revisions will be made to the standards based on data and lessons learned in real world systems and programs. In the short term, our hope is that the availability of standards based on evidence for effectiveness—and shaped by experts from real world systems—will help improve existing programs, establish new ones, and provides adequate funding and other resources to implement the highest-quality IHBT programs possible.

## Appendix

See Tables 5 and 6.

**Table 5:** Final list of program standards for intensive in-home behavioral health treatment (IHBT)

Clinical program categories	Description
(1) Competent staff	1.1 *Role clarity* Regardless of composition of teams (i.e., solo practitioners; two or three-person teams), there are clear roles and responsibilities for the IHBT practitioners, including detailed job descriptions for each role [e.g., therapists, other qualified mental health professionals (QMHPs), peer support workers, supervisors]
	1.2 *Practitioner credentials* All members of IHBT teams (i.e., therapists, QMHPs, peer support workers, supervisors) have a clear set of credentials (i.e., relevant degree, training, and experience) appropriate to their role. Moreover, regardless of composition of teams (i.e., solo practitioners; two or three-person teams), IHBT teams as a whole have credentials that allow them to provide the complete array of services included in IHBT (i.e., if the program utilizes a single practitioner model, staff need to have the necessary credentials to provide the full continuum of services)
	1.3 *Qualified personnel* Practitioners must possess the ability to engage youth and caregivers and build and maintain relationships, as well as demonstrate skills appropriate to their role (e.g., therapist, QMHP, peer support worker, supervisor). Ideally, IHBT practitioners have prior experience and/or training (2000+ h) working with youth with intensive needs and their caregivers. Practitioners with little to no previous experience in IHBT are required, before taking on a full caseload, to shadow a more experienced practitioner or supervisor and practice under observation with feedback until they demonstrate competence
	1.4 *Stable workforce* The organization or team will take specific steps (e.g., evaluate adequacy of compensation and benefits, assess and attend to organizational climate), to ensure that turnover among staff is maintained at a level that does not negatively impact youth and caregivers or detrimentally affect the performance of the IHBT program. Ideally, staff turnover is maintained at < 25% annually
	1.5 *Rigorous hiring processes* The IHBT provider organization has written interviewing and hiring protocols for each of the relevant positions. Protocols are rigorous and aimed at recruiting and selecting staff who are ideal for the position (e.g., presentation of scenarios that allow candidates to demonstrate skills/value base)
	1.6 *Reflective hiring process* The IHBT program undertakes all steps possible to ensure staff recruitment and hiring reflects the racial, cultural, and linguistic diversity of the population(s) being served
	1.7 *Effective training* IHBT staff and supervisors are required to participate in initial training and continuing education relevant to their roles and responsibilities. There are written training protocols that include behavioral rehearsal and direct observation of skills-based practice
	1.8 *Ongoing skills-based coaching* IHBT practitioners have access to regular (at least once a week) clinical supervision or consultation by a supervisor who observes the practitioner’s skills, reviews plans of care and other documentation, and provides feedback aimed at improving practice
	1.9 *Intensive supervision* Supervisor to practitioner ratio meets relevant guidelines (ideally, no higher than 1:8) and supervisors allocate adequate time to the IHBT team (ideally, 50% FTE for up to 4-person team; and 100% for 5–8 person team). In addition, the IHBT supervisor convenes weekly team meetings/group supervision to coordinate treatment, supports, review safety plans, and coordinate crisis on-call between team members
	1.10 *Quality of supervision* Supervisors review initial and updated IHBT treatment plans, treatment notes, and progress for each youth and caregiver as part of the process of overseeing IHBT implementation
	1.11 *On call support* Programs arrange for 24/7 on-call support for their staff
(2) Defined practice model	2.1 *Clear eligibility criteria* Population of focus is limited to youth with intensive behavioral health needs (e.g., multiple diagnoses, multiple action items on a standardized assessment, and/or significant safety or risk concerns) and who are at risk of out-of-home placement or transitioning home from an out-of-home placement due to their behavioral health needs
	2.2 *Practice protocol(s)* A standardized protocol is used by program staff with all youth and caregivers that guides an individualized selection of IHBT interventions to be provided relative to youth and caregivers’ strengths, needs, goals, and preferences
	2.3 *Service coordination* In situations where there is no care coordination program or provider, IHBT provider integrates case/care management into the practice model, including a well-operationalized approach to coordinating and overseeing multiple therapeutic services and supports (formal and informal) for each youth and caregiver
	2.4 *24/7 availability* Ideally, crisis response is available at all times by IHBT program staff. If/when coverage is not available from the IHBT provider, the youth and caregivers have knowledge of and access to services such as Mobile Crisis Response
	2.5 *Commitment to flexibility and accessibility* IHBT sessions are delivered at times and in places that are flexible, accessible, and convenient to the youth and caregivers, including evening and weekend appointment times, and sessions at the location of the youth and caregivers’ choice
	2.6 *Ecological focus* IHBT is based on a holistic and comprehensive assessment of youth and caregiver needs. Treatment planning conceptualizes the youth and caregivers as part of an ecological system
	2.7 *Comprehensiveness of intervention* Whether delivered via one individual or by members of a team, the youth and caregivers have access to comprehensive behavioral health treatment, including, but not limited to: behavioral management training, skills-enhancement, individual therapy, family therapy, psychiatric evaluation and medication management, 24/7 crisis response, family and peer-to-peer support, and care coordination
	2.8 *Safety planning* Safety plans are completed collaboratively with the youth, caregivers and IHBT team, and include: assessment of safety concerns, escalation patterns, and crisis triggers; incorporation of natural supports; actionable crisis stabilization and de-escalation strategies that are easily understood; and prevention strategies. Plans are monitored regularly, with revisions and additions occurring as clinically indicated
	2.9 *Small caseloads* The number of youth and caregivers per practitioner is appropriate to the practice model and intensity (ideally, 6:1, 8:1, and 12:1 for one, two, and three-person teams respectively)
	2.10 *Intensity of intervention* Frequency and hours of intervention are tailored to the level of need for support of the youth and caregivers and their status in the intervention process. Average intensity for youth and their caregivers, however, should be no < 3–6 h per week. In some cases, factors such as phase of treatment may warrant flexibility
	2.11 *Focused treatment duration* Youth and caregivers are engaged in IHBT until intervention goals are met and progress has been documented via ongoing assessment. However, the intervention should aim to address the youth and caregivers’ priority needs and transition out of formal IHBT within 6 months. IHBT provider organizations’ average length of treatment should be 3–6 months
	2.12 *Post-transition services* The program includes a procedure for checking in with the youth and caregivers for 6 months after transition from formal IHBT
(3) Accountability mechanisms	3.1 *Outcome monitoring* Baseline and repeated measurement of outcomes is routinely and reliably measured and shared with the youth and caregivers, including: emotional and behavioral functioning of the youth, living situation, school outcomes, juvenile justice involvement, and progress toward individualized goals for the youth and caregivers. Youth and caregiver satisfaction with the team and process should also be assessed
	3.2 *Quality monitoring* IHBT practice adherence and quality of care is routinely and reliably measured with the goal of providing feedback and opportunity for skill development for the practitioner as well as continuous program improvement
	3.3 *Effective data management* The IHBT provider organization uses information systems that serve as a mechanism for maintaining information for each youth and caregiver. Data systems can generate reports that are routinely used to monitor individual youth and caregiver progress, assist in supervision, and manage the IHBT program
	3.4 *Review of care plans* Each youth and caregiver’s initial treatment plan is reviewed by an expert (i.e., supervisor) in the IHBT practice model. Updated treatment plans should also be regularly reviewed
(4) Leadership	4.1 *Comprehensive system collaboration* The IHBT provider establishes and maintains effective partnerships with community partners including representatives of all child serving systems, caregiver- and youth-run organizations, and other provider organizations. IHBT provides proactive system advocacy for youth and caregivers
	4.2 *Positive work environment* Supervisor and program administrators monitor and address staff morale and encourage a high sense of collective mission, open communication, and cohesion among all staff
	4.3 *Effective leadership* Supervisors and higher-level leadership are receptive to the ideas and concerns of staff, have well-defined organizational performance goals, and effectively address barriers
(5) Facilitative organizational support	5.1 *Adequate compensation* IHBT practitioners and their supervisors are adequately compensated and given the physical resources needed (e.g., office space, laptops, transportation support) to do the job
	5.2 *Routine oversight of key operations* The IHBT organization has individuals responsible for (1) overseeing human resources (i.e., recruitment, training, coaching, performance assessment, staff retention); (2) data collection and use; and (3) IHBT implementation, including review of youth and caregiver enrollment patterns and plans of care

**Table 6:** Final list of practice standards for in-home behavioral health treatment (IHBT)

Clinical practice standards	Description
(1) Engagement	1.1 *Describes IHBT* Ensures that the process of IHBT is described clearly for youth and caregivers, including: roles, boundaries, strengths and limitations, particularly as they differ from other treatment settings and modalities. Orients, in plain language, the expectations of all team members, including youth and caregivers
	1.2 *Explains confidentiality* (and its limitations) specific to the IHBT model, including how and why information may be shared with individuals within the team (e.g., caregivers) and outside the team (e.g., for supervision)
	1.3* Engages the youth/caregivers/family* utilizing evidence-based techniques. These include: A. Promotes youth and caregiver voice and choice B. Identifies potential future barriers to participating in treatment and actively brainstorms solutions C. Reframe or clarify youth and caregiver perspectives in a way that avoids criticism or judgement D. Utilize strength-based language and practices
	1.4 *Employs motivational enhancement strategies* (e.g., open-ended questioning, affirmations, solution-focused, and reflections), based on the youth and caregivers’ readiness for change
(2) Cultural competence	2.1 *Actively seeks to understand* and demonstrate respect for the unique and diverse backgrounds of the youth and caregivers (e.g., roles, values, beliefs, races, ethnicities, sexual orientations, gender expressions, gender identities, languages, traditions, communities, and cultures)
	2.2 *Uses language that is accessible* to the youth and caregivers and, where necessary, translates clinical terminology (e.g., diagnoses and acronyms) used by professionals into content that is clear and promotes understanding
(3) Risk identification	3.1 *Identifies risks* Works with the youth and caregivers to identify and address risk and safety concerns at home, in school, and in the community
(4) Safety planning	4.1 *Co-creates safety plan* Ensures that the youth and caregivers have an individualized safety plan. Safety plans should be co-created by the youth and caregivers, and include the identification of safety concerns, potential crises, triggers, de-escalation and coping strategies utilizing functional strengths, actionable stabilization steps, prevention measures, and youth- and caregiver-identified supports
	4.2 *Regularly monitors and updates the safety plan* in partnership with the youth, caregivers, and other team members
(5) Crisis response and stabilization	5.1 *Responds to crises* Is available as an initial crisis responder, responding to calls immediately and providing on-site stabilization as necessary, depending on the youth and caregivers’ preferences and other options within the system of care. Makes other arrangements for coverage when IHBT team members or supervisor is unavailable (e.g., Mobile Crisis Services)
	5.2 *Uses crisis de-escalation* skills and demonstrates ability to effectively prevent or stabilize crisis situations. Works with the youth and caregivers to develop their own crisis de-escalation skills
(6) Comprehensive contextual assessment	6.1 *Identifies needs and current functioning* Works with youth and caregivers to comprehensively assess current strengths, behavioral health needs, and functioning across key life domains (e.g., school, vocation, family, social, and community)
	6.2 *Identifies functional strengths* Works with the youth and caregivers to identify strengths that can be used as the basis for elements of the treatment plan in the areas of: school, vocational, family, social, and community functioning as well as towards meeting developmental skills/abilities
	6.3 *Assesses for trauma* Assesses for the presence and impact of trauma (e.g., personal, intergenerational, community, and historical) in the youth and caregivers
(7) Clinical conceptualization process	7.1 *Prioritizes needs* Works with the youth and caregivers to prioritize the most critical behavioral health needs and concerns that will be the focus of treatment planning and delivery. If care planning includes other team members, such as in team-based wraparound or other care coordination models, prioritization will be conducted in collaboration with other team members as well
	7.2* Conducts a functional analysis* Works with the youth and caregivers to conduct a functional analysis of antecedents and consequences of the youth's behavior to yield a functional understanding of behavior. Ideally, an external expert (supervisor, coach, or model consultant) reviews and provides feedback on these factors and their applicability to treatment planning
(8) Collaborative treatment planning	8.1 *Develops treatment plan* Works with the youth and caregivers to develop a treatment plan, written in the language of the family, with a manageable number (e.g. 1–4) of priority needs and goals. Strategies for addressing the needs and goals are based use evidence-based techniques wherever appropriate
	8.2 *Develops indicators of progress* Works with the youth and caregivers to develop individualized indicators of progress that are concrete and measurable for each priority treatment need/goal in the plan of care
(9) Psychoeducation	9.1 *Provides psychoeducation* Engages the youth and caregivers in initial and continued psychoeducation surrounding the youth’s diagnoses and/or behavioral health needs, as well as applicable intervention strategies
(10) Measuring and monitoring treatment progress	10.1 *Conducts standardized assessment* Collaborates with youth and caregivers to use BOTH standardized forms of assessment and indicators that are individualized to the youth/caregivers to measure progress from baseline to regular follow-up intervals. Such assessment is also used near treatment plan completion to determine the youth and caregivers’ readiness for discharge
(11) Skill building—youth: functional competencies and coping strategy development	11.1 *Builds youth skills* Works with youth and caregivers to develop adaptive and emotional coping skills across settings, such as emotional regulation, problem solving, communication, conflict management, and decision-making
(12) Skill building—parent: behavior management and positive parent–child relationships	12.1* Builds caregiver skills* Works with caregivers to help them acquire and use behavior management skills as indicated by the treatment plan. Examples include: consistency and follow through, use of meaningful rewards and consequences, problem solving, praise and positive communication, conflict resolution, and the development of child supervision and monitoring plans
	12.2 *Promotes positive relationships* Works with caregivers to develop supportive and nurturing relationships with the youth that promote resiliency and wellness
(13) Cognitive behavioral interventions—youth	13.1 *Uses cognitive-behavioral strategies* Demonstrates competency in cognitive behavioral interventions, including assisting the youth and caregivers in identifying underlying emotions and emotional triggers, and in developing cognitive flexibility, emotional regulation, and/or adaptive thinking patterns
(14) Family and system interventions: structural, solution focused, strategic	14.1* Promotes positive family interactions* Works with the youth and caregivers to identify non-adaptive interactional patterns, and is able to develop and implement family system interventions that increase the youth and caregivers’ adaptive responses and functioning
(15) Collaborative planning and care coordination	15.1* Ensures collaboration* Ensures that there is cross-system collaboration and service coordination, including regular cross-system collaboration meetings
	15.2 *Assesses for substance abuse treatment needs* Works with the youth and caregivers to assess for substance use needs and, if appropriate, integrates treatment into the plan of care (unless assessment indicates a need for a different treatment approach or setting)
	15.3 *Assesses for social service needs* Works with the youth and caregivers to assess for the need of social services (e.g., food subsidies, housing and utilities assistance, and job training) and, if appropriate, engage services
(16) Contextual interventions	16.1 *Promotes positive relations with systems* Supports and empowers the youth and caregivers to develop positive working relationships with other systems and providers who are engaged with the youth and/or caregivers
	16.2 *Arrange for supports from systems* Works with the youth and caregivers to consult with other system providers to develop and implement relational supports and accommodations (e.g., support developing an IEP or 504 plan in school) based on the youth and caregivers’ abilities and challenges
(17) Strategic advocacy	17.1 *Provides system navigation* Works with the youth and caregivers to understand each of the systems they are involved in, as well as shares and models how they can effectively navigate those systems. Where there is a care coordination provider and/or family/youth partner, shares responsibility for system understanding and navigation
(18) Resilience/development asset/wellness promotion	18.1 *Builds community assets* Works with youth and caregivers in linking youth with pro-social activities and peers
	*Builds a future orientation* Works with youth and caregivers to build a future orientation (e.g., optimism and goals)
(19) Resource and support building: identification and linkage	19.1* Identifies current family resources and supports* Works with the youth and caregivers to identify their current support network (informal and formal) across areas such as instrumental (e.g. childcare, transportation, etc.), informational, and emotional supports, as well as the availability of the supports, and the size and stability of the support network
	19.2 *Builds family resources and supports* Works with the youth and caregivers to determine if additional supports are needed. When appropriate, the practitioner helps to facilitate the development and linkage of a safety net of supports for the youth and caregivers
(20) Transition planning	20.1 *Establishes transition criteria* Works with the youth and caregivers, early in the intervention, to develop a plan for transition from IHBT by establishing criteria for successful transition/discharge
	20.2* Develops post-IHBT crisis plan* Works with the youth and caregivers to develop a post-IHBT crisis management plan that includes prevention strategies, action steps, specific responsibilities, and communication protocols
	20.3 *Develops skill maintenance plan* Works with the youth and caregivers to develop a plan for ongoing maintenance of skills and progress
	20.4 *Develops linkages with post-IHBT resources* Works with the youth and caregivers to develop linkages to post-IHBT resources and supports (informal and formal), as appropriate
	20.5* Discusses access to future IHBT services* Discusses with the youth and caregivers how they can access future IHBT services, as needed
(21) Transition	21.1 *Schedules a closing session* to review progress towards meeting needs/goals, celebrate successes, and discuss the youth and caregivers’ experiences of the treatment process
